# Chorea as a clinical feature of the basophilic inclusion body disease subtype of fused-in-sarcoma-associated frontotemporal lobar degeneration

**DOI:** 10.1186/s40478-016-0304-9

**Published:** 2016-04-04

**Authors:** Ito Kawakami, Zen Kobayashi, Tetsuaki Arai, Osamu Yokota, Takashi Nonaka, Naoya Aoki, Kazuhiro Niizato, Kenichi Oshima, Shinji Higashi, Omi Katsuse, Masato Hosokawa, Masato Hasegawa, Haruhiko Akiyama

**Affiliations:** Dementia Research Project, Tokyo Metropolitan Institute of Medical Science, 2-1-6 Kamikitazawa, Setagaya-ku, Tokyo, 156-8506 Japan; Department of Psychiatry, Tokyo Metropolitan Matsuzawa Hospital (TMMH), Tokyo, Japan; Department of Neurology, JA Toride Medical Center, Ibaraki, Japan; Division of Clinical Medicine, Department of Neuropsychiatry, Faculty of Medicine, University of Tsukuba, Ibaraki, Japan; Department of Neuropsychiatry, Okayama University Graduate School of Medicine, Dentistry and Pharmaceutical Sciences, Okayama, Japan; Department of Psychiatry, Yokohama City University, School of Medicine, Kanagawa, Japan

**Keywords:** FTLD-FUS, BIBD, bvFTD, Chorea, Involuntary movement, Parkinsonism

## Abstract

Choreoathetoid involuntary movements are rarely reported in patients with frontotemporal lobar degeneration (FTLD), suggesting their exclusion as a supportive feature in clinical diagnostic criteria for FTLD. Here, we identified three cases of the behavioral variant of frontotemporal dementia (bvFTD) that display chorea with fused in sarcoma (FUS)-positive inclusions (FTLD-FUS) and the basophilic inclusion body disease (BIBD) subtype. We determined the behavioral and cognitive features in this group that were distinct from other FTLD-FUS cases. We also reviewed the clinical records of 72 FTLD cases, and clarified additional clinical features that are predictive of the BIBD pathology. Symptom onset in the three patients with chorea was at 44.0 years of age (±12.0 years), and occurred in the absence of a family history of dementia. The cases were consistent with a clinical form of FTD known as bvFTD, as well as reduced neurological muscle tone in addition to chorea. The three patients showed no or mild parkinsonism, which by contrast, increased substantially in the other FTLD cases until a later stage of disease. The three patients exhibited severe caudate atrophy, which has previously been reported as a histological feature distinguishing FTLD-FUS from FTLD-tau or FTLD-TAR DNA-binding protein 43. Thus, our findings suggest that the clinical feature of choreoathetosis in bvFTD might be associated with FTLD-FUS, and in particular, with the BIBD subtype.

## Introduction

Frontotemporal lobar degeneration (FTLD) is a neurodegenerative disease that commonly causes dementia [1]. In clinical practice, FTLD is considered a syndrome and is presently classified by the consensus criteria of Neary and colleagues [2] into three subtypes: frontotemporal dementia (FTD), progressive nonfluent aphasia, and semantic dementia. Most patients with progressive nonfluent aphasia and semantic dementia show some features of FTD (e.g., behavioral symptoms), later on in their disease course. Those with FTD as the dominant clinical picture in the early disease stage are currently referred to as having a behavioral variant of FTD (bvFTD) [3].

The neuropathology of FTLD is as complex as the clinical syndrome. Virtually all patients with FTLD have abnormal intracellular accumulations of disease-specific molecules. These molecules include tau, TAR DNA-binding protein 43 (TDP-43), and fused in sarcoma (FUS) [4, 5]. FTLD cases are now assigned to one of three major molecular subgroups based on histopathological findings: FTLD-tau, FTLD-TDP, or FTLD-FUS [5]. Before the discovery of TDP-43 in 2006 [6, 7], most cases of tau-negative FTLD were collectively termed FTLD-U because their inclusions were ubiquitin-positive. Subsequently, it became apparent that the majority of FTLD-U cases were in fact FTLD-TDP, (i.e., FTLD with TDP-43 inclusions), with 10 to 20 % of FTLD-U cases remaining as tau-negative and TDP-43-negative FTLD. In 2009, FUS was identified as one of the genes for familial amyotrophic lateral sclerosis (ALS) [8, 9]. Consequently, most tau-negative and TDP-43-negative FTLD inclusions were found to be FUS positive [10–12]. Accordingly, cases of FTLD with FUS-positive inclusions are now collectively called FTLD-FUS. Three rare forms of FTLD are considered to be subtypes of FTLD-FUS: atypical FTLD-U (aFTLD-U), basophilic inclusion body disease (BIBD), and neuronal intermediate filament inclusion disease (NIFID) [11]. Although these three subtypes may represent a continuous spectrum of FTLD-FUS disease, detailed histopathological investigation suggests they are closely related but distinct entities [11–13].

Several previous reports have challenged these clinicopathological relationships in FTLD patients. In FTLD-FUS, which is present in a minority of FTLD patients, such relationships have only recently been described [12, 14–19]. These studies reveal that FTLD-FUS patients may have a relatively younger onset (often before the age of 40 years), absence of a family history of the disease, and severe caudate atrophy on imaging [16–18]. Recently, Snowden et al. suggested that aFTLD-U is associated with a cognitive and behavioral phenotype that is distinct from the other forms of FTLD-FUS (specifically, NIFID and BIBD). They noted that aFTLD-U is characterized by prominent obsessiveness, repetitive behaviors and rituals, social withdrawal and lack of engagement, hyperorality with pica, and marked stimulus-bound behavior (e.g., utilization behavior). Furthermore, they suggested that clinical presentation of FTLD with associated FUS pathology may not be related to mutation of the *FUS* gene. Additionally, a uniform clinical phenotype of BIBD and NIFID has been reported in a few studies [12, 15]. Yokota et al. found that NIFID and BIBD share several clinical features including dysarthria, motor neuron signs, parkinsonism, and memory impairment [15]. They also noted that it is difficult to differentiate BIBD from NIFID in clinical practice. While these reports indicate variations in behavioral and cognitive features of FTLD-FUS, the features that are distinct from the other forms of FTLD (i.e., FTLD-tau and FTLD-TDP) remain to be clarified.

Patients with FTLD often report associated motor system impairments, such as parkinsonism and motor neuron disease [2, 20], whereas association of FTLD with chorea and athetosis has rarely been reported. In the clinical diagnostic criteria for FTLD [2], choreoathetosis is one of the diagnostic exclusion features. Chorea is an abnormal involuntary movement characterized by excessive, spontaneous movements that are irregularly timed, nonrepetitive, randomly distributed, and abrupt in character [21]. The classical form of chorea occurs in Huntington’s disease (HD), an inherited neurodegenerative disease in which atrophy of the striatum is the predominant pathology. However, the striatum is also severely affected in all subtypes of FTLD. The results of a recent report found significantly greater striatal atrophy by magnetic resonance imaging (MRI) in FTLD-FUS patients than in FTLD-TDP and FTLD-tau patients, thereby distinguishing FTLD-FUS from other forms of FTLD [16].

Here, we identified three cases of bvFTD with chorea, which were diagnosed as FTLD-FUS and exhibit histopathological results indicative of the BIBD subtype. We identified the behavioral and cognitive features that distinguish this group from other FTLD-FUS cases. Further, we also reviewed the clinical records of 72 FTLD cases to identify distinct clinical features that are predictive of FTLD-FUS, and in particular the BIBD subtype.

## Materials and methods

### Participants

Seventy-two FTLD cases were registered in the autopsy archives of Dementia Research Project, Tokyo Metropolitan Institute of Medical Science, Tokyo, Japan. The molecular pathology observed in these cases is summarized in Table 1. Briefly, the archives included 29 FTLD-tau, 32 FTLD-TDP, and 10 FTLD-FUS cases, and 1 unclassifiable case. Of these, clinical features corresponding to bvFTD with chorea were identified in three cases. The pathological diagnosis in these three cases was BIBD. Case 1 was extensively analyzed neurologically, neuroradiologically and genetically, whereas cases 2 and 3 were reviewed using their clinical records.

**Table 1:** Molecular pathology of 72 FTLD cases

Diagnosis	No. of patients
*FTLD-tau* (29)	
Pick’s disease^a^	18
FTDP-17tau	1
CBD^b^	4
PSP^b^	4
Unclassifiable	2, [53]
*FTLD-TDP* (32)^c^	
Type A	4
Type B	17
Type C	10
Unclassifiable	1, [54]
*FTLD-FUS* (10)	
BIBD	6
NIFID	2
aFTLD-U	1
Unclassifiable	1, [55]
*Unclassifiable* (1)	

*CBD* corticobasal degeneration, *PSP* progressive supranuclear palsy, *FTDP-17tau* frontotemporal dementia with parkinsonism linked to chromosome 17 associated with tau pathology, *BIBD* basophilic inclusion body disease, *NIFID* neuronal intermediate filament inclusion disease; *aFTLD-U* atypical FTLD with ubiquitinated inclusions

^a^Pick’s disease refers to only FTLD-tau with Pick bodies

^b^Cases with CBD and PSP were included only if the patients presented with features of FTLD, such as frontotemporal dementia, semantic dementia and progressive nonfluent aphasia

^c^FTLD-TDP cases were classified using the system reported by Mackenzie et al. [56]

### Neuropathological examination

Brain and spinal cord tissue were fixed in 10 % formalin and embedded in paraffin. Sections (10 μm thick) were cut from the cerebrum, midbrain, pons, medulla oblongata, cerebellum, and spinal cord. The sections were stained with hematoxylin and eosin as well as Klüver–Barrera stain. Immunohistochemistry was performed for tau (AT8, 1:1,000; Thermo Scientific), α-synuclein (pSyn#64, 1:1,000; Wako), TDP-43 (409/410, 1:1,000; original antibody [22]), FUS protein (HPA008784, 1:1,000; Sigma-Aldrich; and A300-302A, 1:500–1,000; Bethyl Laboratories), Ewing sarcoma protein (EWS, 1:100; Santa Cruz Biotechnology), and TATA-binding protein-associated factor 15 (TAF15, 1:50; Bethyl Laboratories). Primary antibody labelling was visualized using 0.2 % 3,3′-diaminobenzidine as the chromogen in combination with an Envision Plus kit (Dako Japan, Tokyo), according to the manufacturer’s instructions.

### Immunoblot analysis

Fresh frozen samples for immunoblot analyses were prepared as previously described [23, 24]. Briefly, frozen brain frontal cortex tissue was obtained from one case each of BIBD (case 1), NIFID (case 8), aFTLD-U (case 9), and a normal control.

Brain tissue was homogenized in 20 volumes (w/v) of homogenization buffer (10 mM Tris-HCl, pH 7.4, 0.8 M NaCl, 1 mM EGTA, and 10 % sucrose). Homogenates were incubated at 37 °C for 30 min in homogenization buffer containing 2 % Triton X-100, and centrifuged at 20,000 × *g* for 10 min at room temperature. Supernatants were further ultracentrifuged at 100,000 × *g* for 20 min. After ultracentrifugation, the resulting supernatants and pellets were recovered for immunoblotting analysis as Triton X-100 soluble and insoluble fractions, respectively. For immunoblotting, primary antibodies for the FUS protein were obtained from Sigma-Aldrich (HPA008784) and Bethyl Laboratories, Inc. (A300-302A).

### Statistical analysis

Fisher’s exact probability test was used to determine the significance of differences in variables, including the frequency of each clinical feature. Values of *P* < 0.05 were accepted as significant. All statistical analyses were performed using GraphPad Prism 4 software (GraphPad Software, USA). Statistical significance of the concentration of involuntary movement in the BIBD subtype was assessed by direct calculation of probability under an assumption of independence in all FTLD cases.

## Results

### Clinical findings

#### Case 1

**Fig. 1:**
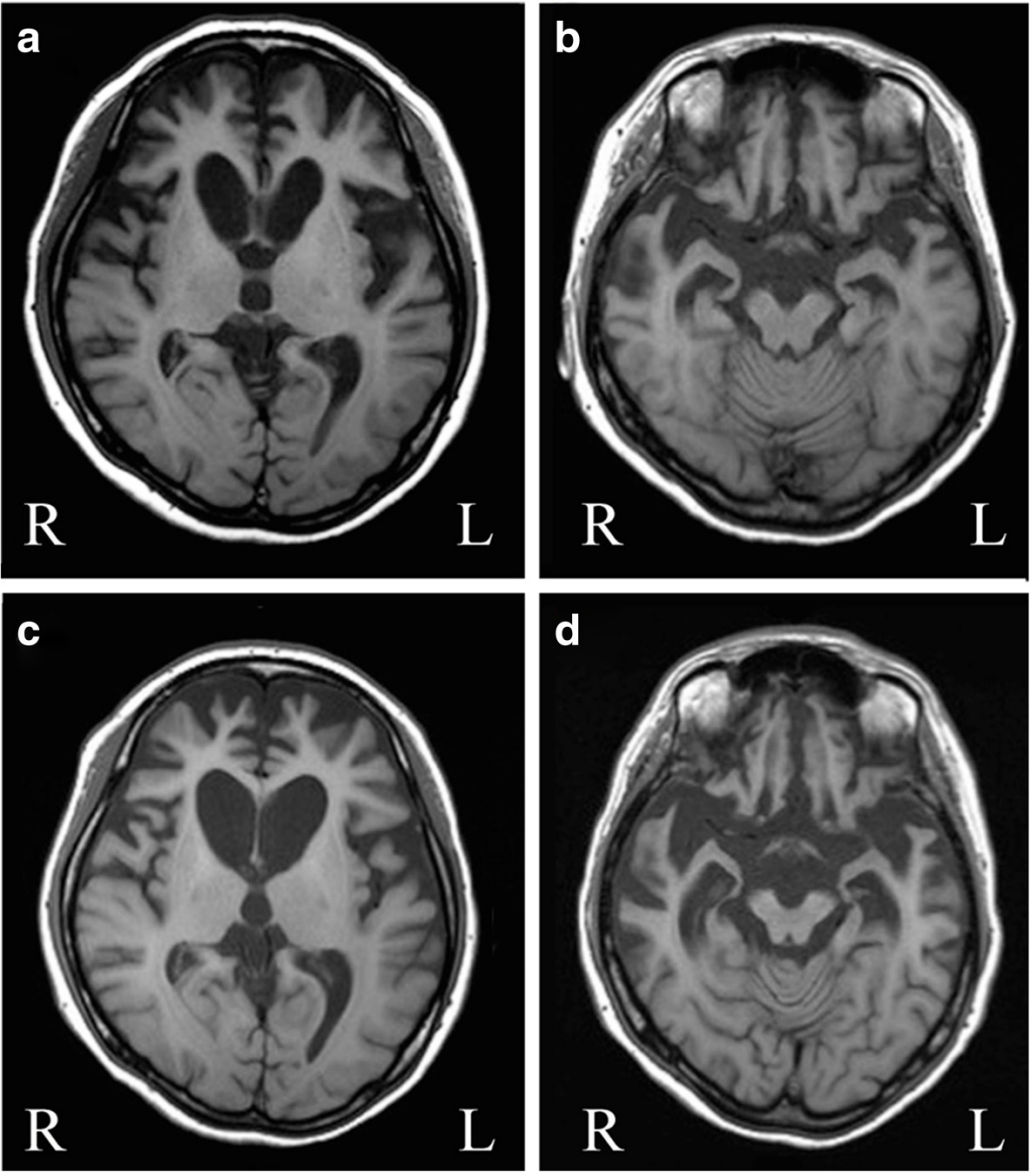
Brain MRI of case 1. **a** and **b** Brain magnetic resonance (MR) images of the patient 1 at age 36. Atrophy is present in the frontal and temporal cortices and the caudate nucleus. **a** and **b** Brain MR images of the patient taken 1 year after those shown in **c** and **d**. The atrophy is more severe and the anterior horn of the lateral ventricles is markedly enlarged. Permission was obtained from the right holder [25]

The patient was a Japanese woman with a family history of schizophrenia but no dementia or movement disorders. She had been living outside of Japan for several years when she developed affective incontinence at the age of 32. Two years later, she was spending much of the day in bed, and displayed palilalia and hyperphagia of carbohydrates such as rice and noodles. She dressed in a provocative manner and was often arrested for shoplifting, but showed no remorse. She was initially diagnosed as having schizophrenia or depressive disorder, and consequently treated with fluvoxamine and olanzapine for a short duration, as well as with electroconvulsive therapy. However, she was unresponsive to these therapies and they were discontinued. At the age of 36, she returned to Japan and was admitted to a psychiatric hospital. She presented with chorea-like involuntary movements of the face, tongue, neck, and four extremities. The involuntary movements included frequent jerking of the shoulders, continuous movement of facial muscles (e.g., lifting the eyebrows, closing the eyes, and thrusting out the tongue), and large amplitude movements of the lower limbs, sometimes with a violent, flinging or flailing quality, which was regarded as ballismus. She also had athetosis-like movements in her right leg. When she wandered the hospital ward, she touched and tapped yellow things. Neurological examination revealed reduced muscle tone, but no muscle weakness, atrophy, or other signs of motor neuron disease. Her speech output was reduced, but she recognized some simple words. Her behavior was stereotyped and ritualistic. Her blood biochemistry test results were normal, including ceruloplasmin and ferritin levels and tests for syphilis. Cerebrospinal fluid concentration of amyloid β-protein, total tau, and phosphorylated tau were normal. Brain MRI results revealed bilateral progressive atrophy in the frontal and temporal cortices and the caudate nucleus (Fig. 1). Hypoperfusion was apparent in these regions by cerebral blood flow single-photon emission computed tomography. The results of an electromyographic investigation were normal. Her Mini-Mental State Examination score was 18/30. She was clinically suspected of having HD because of her chorea and the severe caudate atrophy apparent on MRI imaging. However, she had no repeat expansion in the genes causing HD, spinocerebellar ataxia type 17, or dentatorubral-pallidoluysian atrophy. No mutation was found in the genes for tau, TDP-43, FUS, granulin, amyloid precursor protein, presenilin-1, or presenilin-2. Based on clinical findings, she was diagnosed with bvFTD [2]. She took milnacipran hydrochloride (100 mg) for therapy. At age 37, 5 years after symptom onset, her speech output was reduced and she had dysphagia. Her gross involuntary movements were less severe, but slow and continuous leg movements persisted. At that time, neurological examination showed the presence of primitive reflexes such as sucking, as well as palmomental and strong grasp reflexes bilaterally. She was in a persistent vegetative state by the age of 38. She exhibited arm contractures, but athetosis-like movements (such as the slow, sinuous, continuous flowing external and internal rotation of her right leg) continued until her death. She died of bronchopneumonia at age 39. Her disease duration was 7 years.

#### Case 2

The second patient was a 47-year-old Japanese woman. She had no family history of behavioral change or dementia. She presented with indifference and disinhibition at age 44. She showed polyphagia and subsequent rapid weight gain. She was admitted to a psychiatric hospital at age 46 because of purposeless wandering. She presented with reduced speech output, simple language with stereotypies, and perseveration. She spoke only in uncomplicated, short sentences, but showed no impaired verbal comprehension. Neurological examination revealed continuous and quick chorea-like involuntary movements in the tongue, but no muscle atrophy. She was not administered any medication before presentation of chorea. Her Wechsler Adult Intelligence Scale score was 60 (verbal IQ, 72; performance IQ, 55). Her blood biochemistry results were normal, including a syphilis test. Disorientation or memory impairment were not evident. She showed stereotyped behaviors, such as repeatedly throwing a lot of toilet paper into the toilet. Chorea in her tongue diminished gradually until age 47. She died suddenly of suffocation by food during a hospital stay. Her disease duration was 3 years.

#### Case 3

The third patient was a 67-year-old Japanese woman. There was no relevant family history of dementia, but her sister had died of a nonspecific psychiatric disease. At age 56, she began collecting elastic bands and trash, and eating only rice and pickles. At age 58, she was admitted to a psychiatric hospital because she was drinking a lot of alcohol and committing criminal acts, such as shoplifting. She showed severe impairment of recent memory and disorientation. The results of a neurological examination revealed no abnormalities. Her blood and urine biochemistry results were normal. Behavioral and verbal stereotypy developed gradually. At age 60, she was mute and gradually became bedridden. Contractures in all four extremities were apparent. At age 65, 9 years after symptom onset, rapid and small chorea-like involuntary movements in her neck, trunk, and four extremities became apparent and progressively worsened. Athetosis-like movements were also observed in her left upper extremity. Her chorea-like movements continued until her death, which was due to cardiac failure at age 67. We were unable to obtain drug history data for this case. Her disease duration was 12 years.

### Clinical summary of cases

#### Demographic data

The clinical features of the three patients with chorea and the other FTLD-FUS cases are summarized in Table 2. More detailed clinical descriptions are provided in Japanese (with English abstracts) for cases 1 [25], 2 [26], and 3 [27]. The three patients with chorea had no family history of dementia. Mean age at symptom onset (44.0 ± 12.0 years) for these patients was significantly younger than for other FTLD patients (54.9 ± 18.8 years) obtained from a consecutive clinical cohort in our archives.

**Table 2:** Clinical features of FTLD-FUS

	BIBD						NIFID		aFTLD-U	Unclassified
	〈with chorea〉			〈without chorea〉						
Case No.	1	2	3	4	5	6	7	8	9	10
Sex	F	F	F	M	M	M	F	M	M	F
Onset (age)	32	44	56	57	40	34	67	29	39	30
Duration (y)	7	3.3	12	6	7	6.3	5.7	8	13	15
Family history	No	No	No	No	No	No	No	No	No	No
Initial symptoms	Apathy, Behavioral abnormality	Apathy, Polyphagia, Disinhibition	Behavioral abnormality, memory impairment	Obsessive behaviors	Disinhibition	Weakness in the left hand, dysarthria	Dysarthria	Disinhibition	Apathy, Behavioral abnormality	Behavioral abnormality, memory impairment
Prominent features	Behavioral abnormality	Behavioral abnormality	Behavioral abnormality	Behavioral abnormality	Behavioral abnormality	Motor neuron signs	Pseudobulbar palsy, nonfluent aphasia	Behavioral abnormality	Behavioral abnormality	Behavioral abnormality
Clinical diagnosis	bvFTD	bvFTD	bvFTD	bvFTD	bvFTD	ALS with dementia	CBD	bvFTD	bvFTD	bvFTD
Psychiatric and behavioral symptoms										
Apathy	+	+	+	+	+	+	+	-	+	+
Disinhibition	+	+	+	-	+	-	+	+	+	+
Stereotypy	+	+	+	+	+	-	+	+	+	+
Wandering	+	+	-	-	-	-	+	+	+	+
Altered dietary habits, polyphagia	+	+	+	-	-	-	+	+	+	-
Memory impairment	+	+	+	+	-	-	-	-	+	+
Hypersexuality	+	+	-	-	+	-	+	+	+	-
Oral tendency	+	+	-	+	-	-	-	+	+	-
Perseverations	+	+	+	?	-	?	+	?	-	-
Language and speech										
Reduced speech output	+	+	+	+	+	+	+	+	+	+
Dysarthria	-	-	-	-	-	+	-	-	-	+
Verbal stereotypies	+	-	+	+	-	?	+	?	+	?
Semantic errors	-	-	-	?	-	?	-	?	-	-
Echolalia	+	+	+	?	?	?	+	?	-	-
Neurological signs										
Involuntary movement										
Onset (age)	36	46	65	-	-	-	-	-	-	-
Chorea	+	+	+							
Athetosis	+	-	+							
Ballismus	+	-	-							
Lack of normal muscular tonus	+	+	+	-	-	-	-	-	-	-
Upper motor signs	-	-	-	-	-	+	+	+	-	-
Lower motor signs	-	-	-	-	-	+	+	-	-	-
Dysphagia	-	-	-	-	+	+	+	+	+	+
Gait disturbance	+	+	+	+	+	+	+	+	+	+
Parkinsonism-early	No	No	No	Moderate	No	Moderate	Mild	No	No	Mild
Parkinsonism-late	No	No	Mild	Severe	Severe	Severe	Severe	Severe	Moderate	Severe
Investigations										
CT/MRI (atrophy)	FT(L = R)	na	na	na	FT(L = R)	na	FT(L > R)	na	FT(L = R)	na
EEG	Normal	Slow dominant rhythm	Slow dominant rhythm	na	Normal	na	Normal	na	Slow dominant rhythm	na
EMG	Normal	na	na	na	na	na	na	na	na	na

+, present; -, absent; na, not available

#### Psychiatric, behavioral, cognitive, and language disturbance characteristics

The most frequent initial symptoms in the patients with chorea were apathy and behavioral abnormalities, such as criminal behavior and loss of manners, followed by polyphagia, disinhibition, and memory impairment. All three patients presented with a behavioral abnormality as the prominent feature during the disease course, and were finally diagnosed with bvFTD. Apathy, disinhibition, stereotypy, altered dietary habits, perseveration and memory impairment were the most prominent clinical features in this group. Wandering, hypersexuality, and an oral tendency were observed in two cases. Regarding language disturbances, echolalia and reduced speech output were observed in all three patients, and verbal stereotypies in two. No dysarthria or semantic errors were recognized in this group.

#### Neurological signs

The patients with chorea exhibited reduced muscle tone in addition to chorea. The chorea was complicated by athetosis in two cases and ballismus in one. The three patients lacked parkinsonism signs, even at the later disease stage. In contrast, other FTLD-FUS patients, including BIBD cases without chorea, always showed moderate to severe parkinsonism at the same disease stage. Gait disturbance was observed in all patients. Additionally, the three patients showed neither dysphasia nor upper and lower motor neuron signs, which were noted in some BIBD patients without chorea during the disease course.

#### BIBD compared with all FTLD cases

The demographic data and major clinical features of all our archived FTLD cases are summarized in Table 3. The prevalence rate of FUS pathology in the FTLD cases in our cohort was 13.9 %, which is higher than previously reported (5 %) [28]. Compared with the other subtypes of FTLD-FUS, FTLD-TDP, and FTLD-tau, the most prominent feature in BIBD patients was choreoathetoid involuntary movements, with its occurrence converging at BIBD in all FTLD patients (0.0003 < 0.05). No significant differences were noted for the other clinical features.

**Table 3:** The demographic data and major clinical features of all FTLD cases

	FTLD-FUS				FTLD-TDP				FTLD-tau					Unclassified
	BIBD (*n* = 6)	NIFID (*n* = 2)	aFTLD-U (*n* = 1)	Unclassified (*n* = 1)	Type A (*n* = 4)	Type B (*n* = 17)	Type C (*n* = 10)	Unclassified (*n* = 1)	Pick (*n* = 18)	FTDP-17 (*n* = 1)	CBD (*n* = 4)	PSP (*n* = 4)	Unclassified (*n* = 2)	(*n* = 1)
Sex [male/female(%)]	3/3 (50.0)	1/1 (50.0)	1/0 (100.0)	0/1 (0)	2/2 (50.0)	7/10 (41.2)	6/4 (60.0)	1/0 (100.0)	10/8 (55.6)	1/0 (100.0)	1/3 (0.25)	3/1 (0.75)	0/2 (0)	1/0
onset (y)	49.5 ± 12.1	48 ± 26.9	39	30	53 ± 16.4	58.9 ± 11.0	55.6 ± 3.5	75	56.5 ± 10.9	51	61 ± 3.4	52.5 ± 10.6	53 ± 5.7	46
duration (y)	8 ± 4.6	6.9 ± 1.6	13	15	9.5 ± 7.9	2.6 ± 1.4	12.5 ± 4.8	5.1	8.3 ± 4.5	8	5.5 ± 2.6	13 ± 7.1	6.5 ± 2.1	3.1
clinical diagnosis as bvFTD[n(%)]	5(83.3)	1(50.0)	1(100.0)	1(100.0)	3(75.0)	2(11.8)	5(50.0)	0(0)	12(66.7)	1(100.0)	3(75.0)	1(25.0)	1(50.0)	1(100.0)
involuntary movements[n(%)]	3(50.0)	0(0)	0(0)	0(0)	0(0)	0(0)	0(0)	0(0)	0(0)	0(0)	0(0)	0(0)	0(0)	0(0)
mortor signs[n(%)]	1(16.7)	2(100.0)	0(0)	0(0)	3(75.0)	7(41.2)	7(70.0)	0(0)	2(11.1)	0(0)	0(0)	0(0)	1(50.0)	0(0)

### Neuropathological findings

**Fig. 2:**
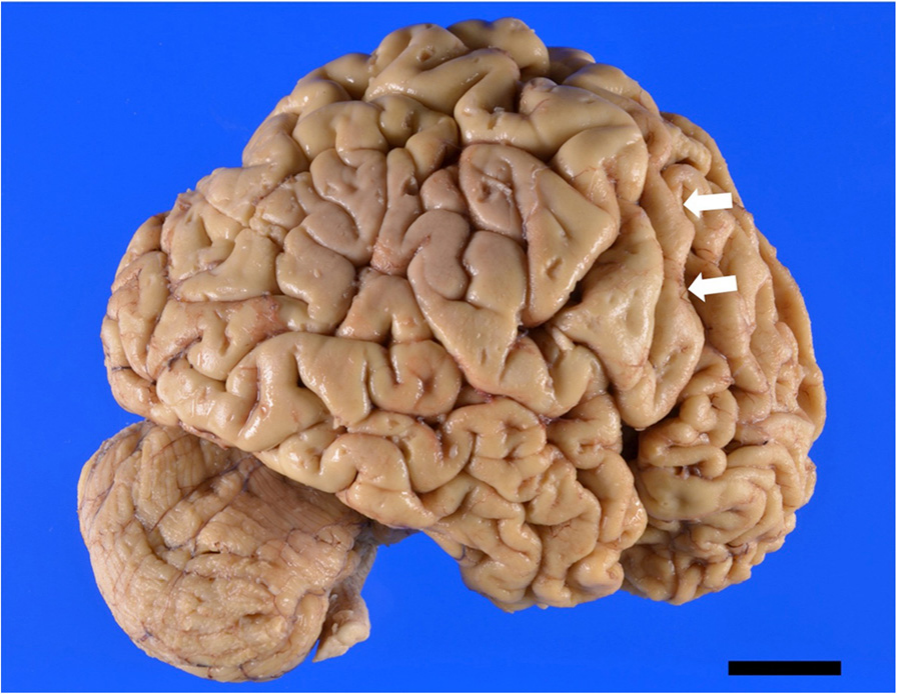
Macroscopic photograph of patient 1. The right hemisphere of the patient is shown. Severe atrophy is present in the frontal cortex and temporal tip. White arrows indicate the precentral gyrus. Scale bar, 2 cm

**Fig. 3:**
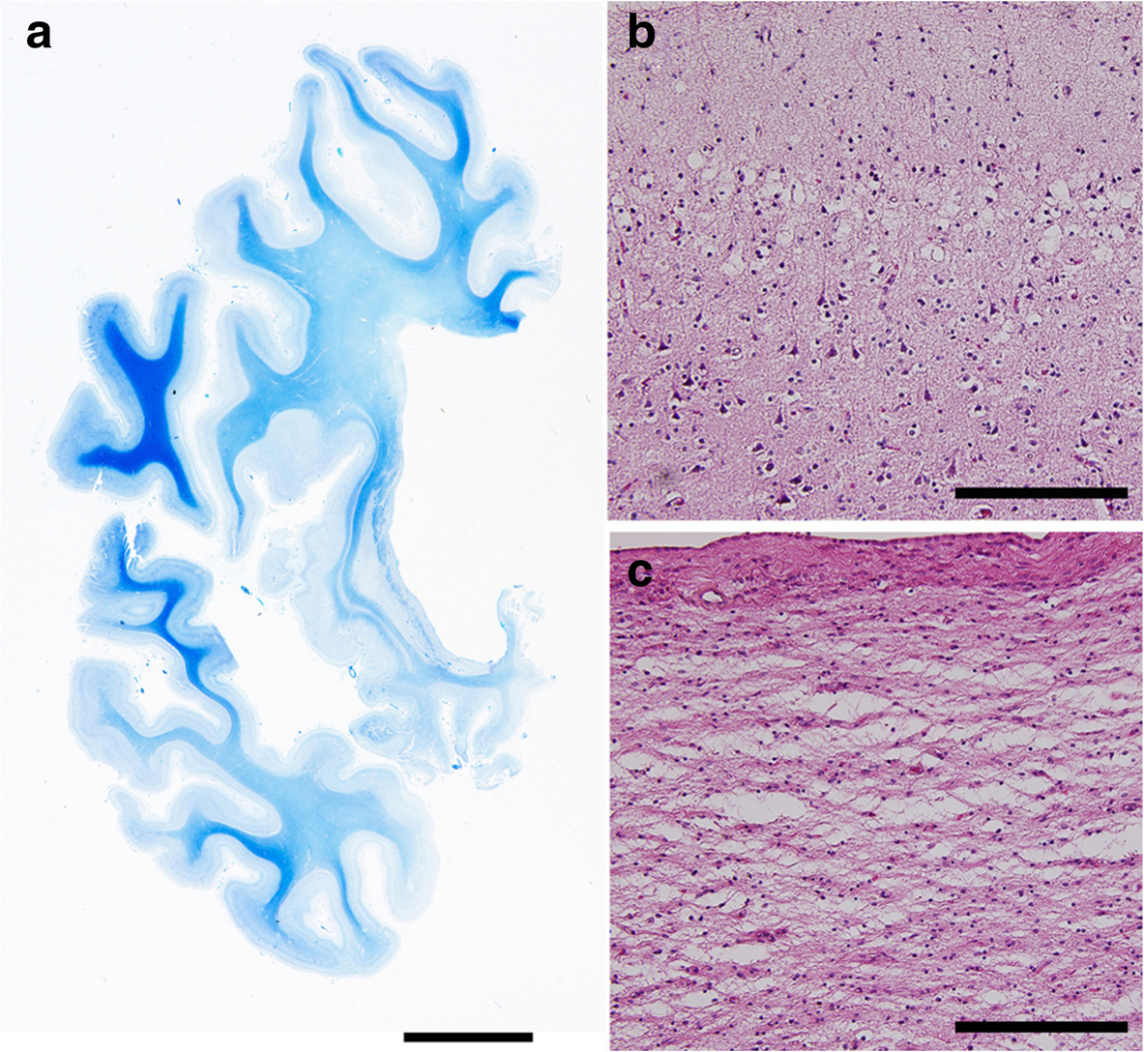
Neuropathological findings of case 1. **a** Semi-macro photograph of the right hemisphere showing severe atrophy of the frontal cortex and caudate nucleus (Klüver–Barrera stain). **b** Severe neuronal loss and astrocytosis in the supragranular layer of the frontal cortex (hematoxylin and eosin stain). **c** Marked neuronal loss and astrocytosis with tissue rarefarction in the caudate head (hematoxylin and eosin stain). **a**–**c** are from case 1. Scale bars, 1 cm (**a**); 200 μm (**b** and **c**)

**Fig. 4:**
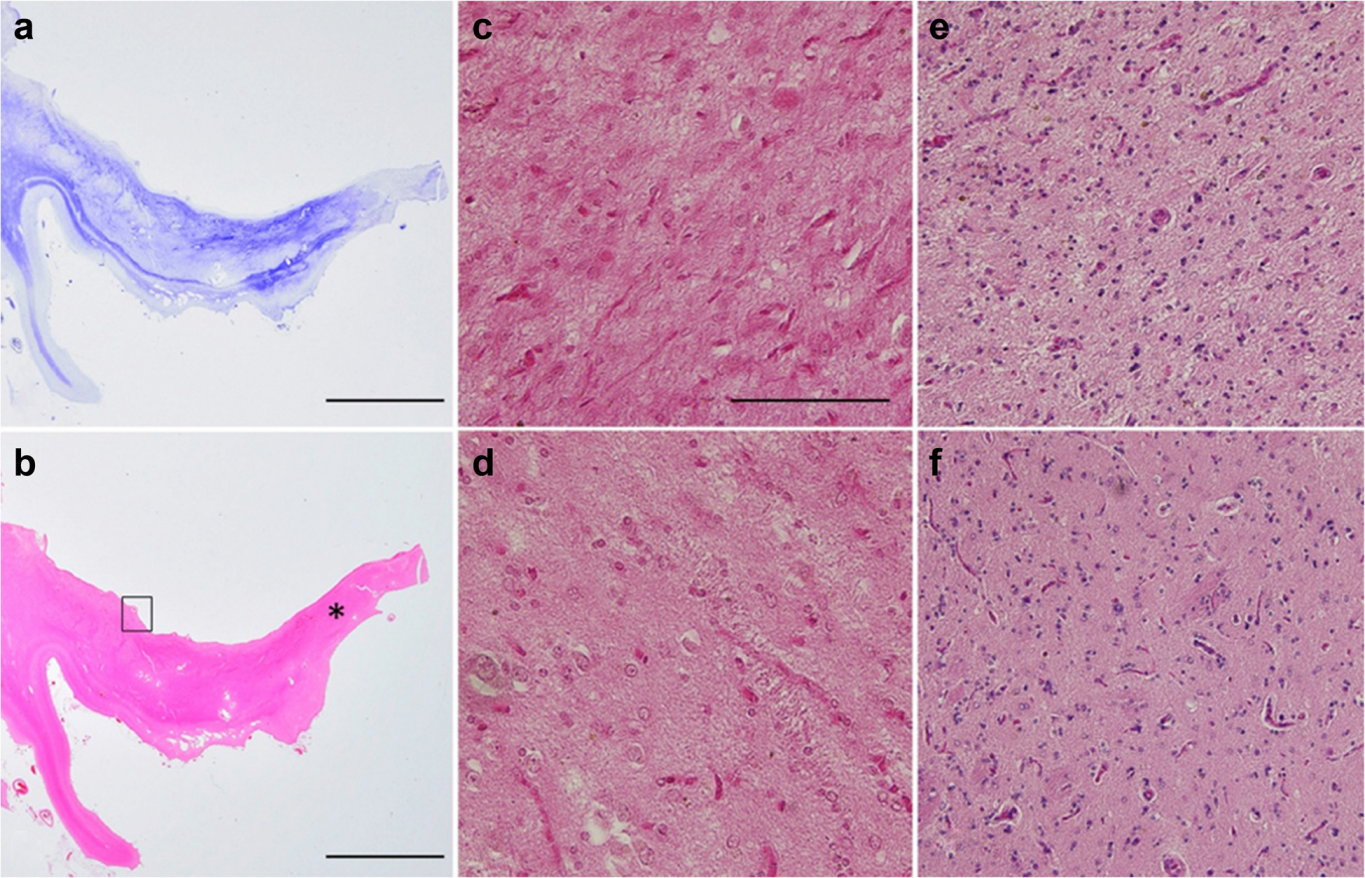
Neostriatum of FTLD-FUS cases. **a** and **b** Semi-macro photograph of the rostal neostriatum including the caudate nucleus head (rectangle), nucleus accumbens (asterisk), and putamen. Severe gliosis (Holzer stain) (**a**) and atrophy (hematoxylin and eosin stain) (**b**). **c** Marked neuronal loss and astrocytosis in the magnified area of the nucleus accumbens (shown by asterisk in **b**). **d** Severe neuronal loss with astrocytosis in the magnified area of the caudate nucleus (shown by the rectangle in **b**). In FTLD-FUS cases, the ventral putamen (**e**) is more involved than the dorsal region (**f**)

The atrophy and degeneration distribution has been described previously for cases 2 and 3 [24, 29]. Here, Table 4 shows the degree and distribution of neurodegeneration in all BIBD cases. Macroscopically, severe frontal cortical atrophy was a consistent finding (Fig. 2 shows case 1). Moreover, the degree of temporal cortical atrophy varied among cases. The caudate nucleus showed marked flattening (Fig. 3a), while pigmentation in the substantia nigra and locus coeruleus was decreased. Histopathologically, neuronal loss and gliosis were prominent in all cases in the frontal cortex and caudate nucleus (Fig. 3b and c). In the striatum, both large and small neurons were severely affected. Along the coronal (or dorsoventral) axis of the neostriatum, ventral striatal regions were more affected than dorsal ones (Fig. 4e and f). Along the mediolateral axis, the paraventricular region of the caudate nucleus was more affected than the paracapsular region. The nucleus accumbens was severely affected (except for one case: case 2). Although the degree and distribution of neurodegenerative changes showed some variance among cases, alterations in no specific region appeared related to the presence or absence of chorea.

**Table 4:** Distribution and severity of neurodegenerative changes in patients with BIBD

	Cases with chorea			Cases without chorea		
	1	2	3	4	5	6
Brain weight (g)	800	1190	880	1140	940	1230
Cortical atrophy	F, T(base)	F(tip)	F&T(base)	F&T(base)	F,T(base)	T(tip)
Frontal cortex	+++	+++	++	+++	+++	+++
Cingulate gyrus	+++	+++	+++	+++	+++	+++
Temporal cortex	++	+	++	++	+++	+++
Hippocampus	+++	+	+++	+++	na	+++
Amygdala	+++	+	na	+++	+++	+++
Caudate nucleus	+++	+++	+++	+++	+++	+++
Putamen	+++	+	+++	++	+++	+++
Globus pallidus	+++	±	++	++	++	++
Thalamus	+++	±	±	++	+++	+
Subthalamic nucleus	±	±	±	±	±	±
Nucleus basalis of Meynert	+	-	±	±	±	+
Cerebellar dentate nucleus	±	-	±	±	±	+
Red nucleus	±	±	±	na	na	±
Substantia nigra	+++	+	+++	++	+++	+++
Locus coeruleus	+++	-	±	±	++	++
Pontine nucleus	±	±	±	±	±	±
Dorsal vagal nucleus	±	±	±	na	±	±
Hypoglossal nucleus	±	-	±	±	±	±
Inferior olivary nucleus	±	±	±	±	+	+
Spinal cord	-	-	na	na	na	±

Data for cases 2 through 6 were assembled from previously published materials [15, 29]. F, frontal lobe; T, temporal lobe; F(tip), frontal tip; F(base), frontal base, T(tip), temporal tip; T(base), temporal base. Rating for the severity of degeneration: -, no degeneration; ±, no neuronal loss but gliosis; +, slight neuronal loss and gliosis; ++, moderate neuronal loss and gliosis; +++, severe neuronal loss and gliosis. Degeneration in the corticospinal tract: +, present; -, absent. na, tissue not available

**Fig. 5:**
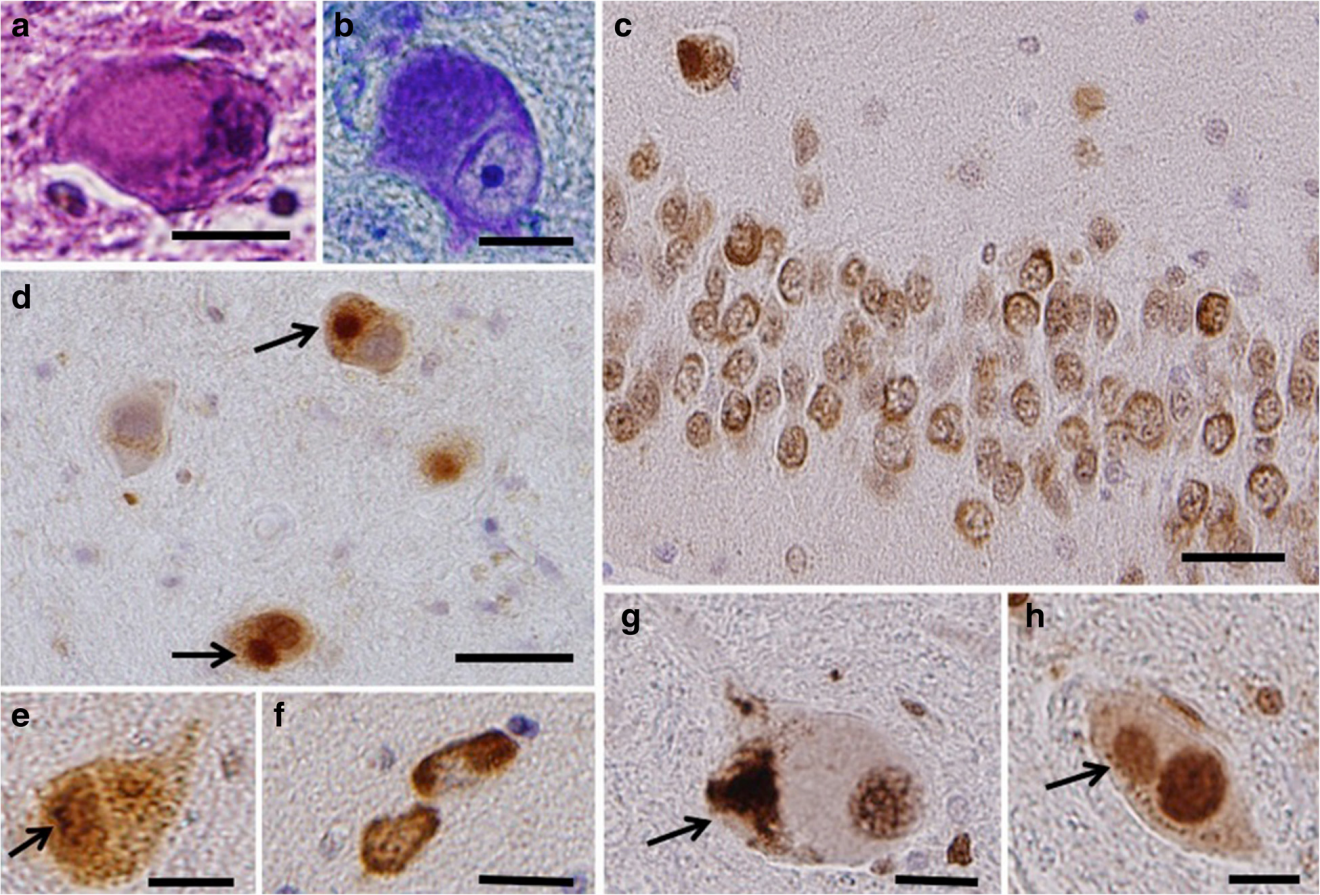
Inclusions detected in patients. Inclusions observed in cases 1 (**a**, **e**, **f, g** **, h**), 2 (**c, d**), and 3 (**b**). **a** A basophilic inclusion in the pontine nucleus (hematoxylin and eosin stain). **b** A basophilic inclusion in the inferior olivary nucleus (Klüver–Barrera stain). **c**, **d**, **e ** and **f** FUS-immunopositive labelling of neuronal cytoplasmic inclusions (NCI) in the hippocampal dentate gyrus (**c**), inferior olivary nucleus (**d,** **e**) and caudate nucleus (**f**).**g** and **h** NCI in the thoracic spinal cord are immunopositive for TATA-binding protein-associated factor 15 (**g**) and Ewing sarcoma protein (**h**). Scale bars: 10 μm (**a**, **b**, **d**, **f**, and **h**); 30 μm (**c**); 15 μm (**e**)

In all BIBD cases, neuronal cytoplasmic inclusions (NCI) were slightly basophilic (Fig. 5a and b) and immunopositive for FUS (Fig. 5c–f). FUS-immunopositive NCIs were distributed in the cerebral cortices, hippocampus (Fig. 5c), the basal nuclei (Fig. 5f), brain stem nuclei (Fig. 5d and e), and spinal cord (in cases where tissue was available). Morphology of the NCIs was variable, and either round, crescent, or annular. Furthermore, NCIs were also immunopositive for EWS (Fig. 5h) and TAF15 (Fig. 5g), but immunonegative for tau, α-synuclein, and TDP-43. The morphology and distribution of NCIs were consistent with those previously described for BIBD [12, 13, 30]. BIBD cases had numerous basophilic inclusions compared with other types of FTLD-FUS. No BIBD (except for case 1) aFTLD-U, or unclassifiable cases had intermediate filament-immunoreactive NCIs. The occurrence of NCIs did not distinguish between the presence or absence of chorea.

### Biochemical findings

**Fig. 6:**
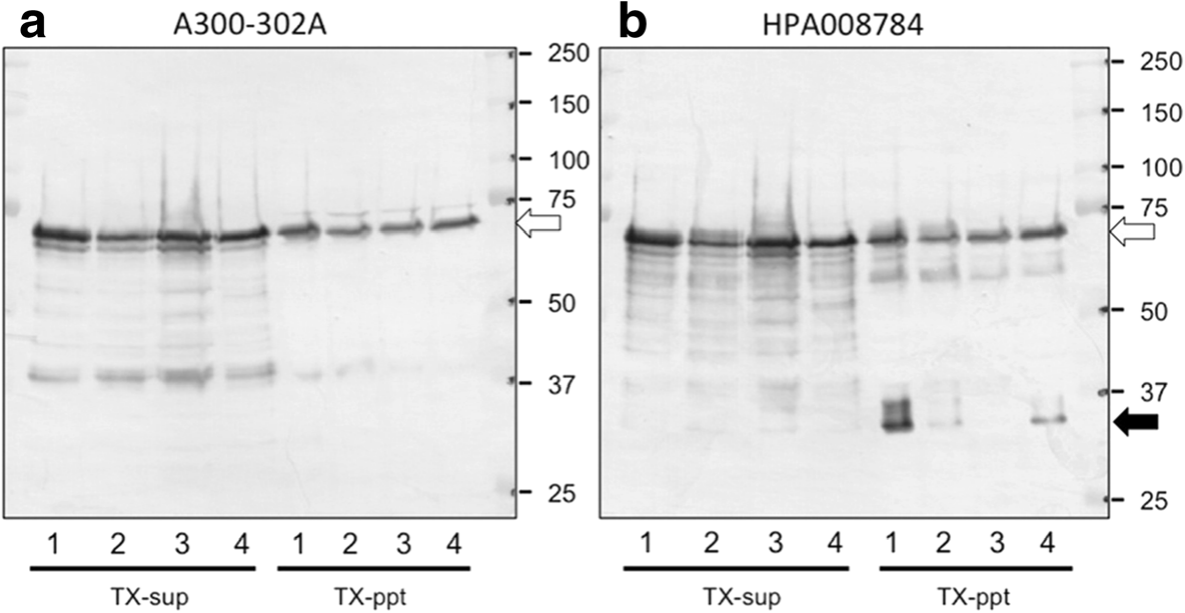
Biochemical analysis of FUS. Proteins were sequentially extracted from brains of patients with BIBD, aFTLD-U, NIFID, and from a control subject. Fractions from patients diagnosed as BIBD with chorea (case 1, lane 1), aFTLD-U (case 9, lane 2), or NIFID (case 8, lane 3) and the control patient (lane 4) were separated by 7.5 % SDS-PAGE and immunoblotted with anti-FUS antibodies (**a** A300-302A; **b** HPA008784). All cases, including the control, show a strong 73-kDa band (*white arrow*) in both soluble and insoluble high-salt fractions. Additionally, the BIBD with chorea patient has a strong band at approximately 33 kDa (*black arrow*; **b** TX-ppt, lane 1), which is less prominent in the other samples

Triton X-100 soluble and insoluble brain fractions extracted from patients diagnosed as BIBD with chorea, aFTLD-U, or NIFID, as well as from a control patient were separated by 7.5 % sodium dodecyl sulfate polyacrylamide gel electrophoresis (SDS-PAGE) and immunoblotted with an anti-FUS antibody (Fig. 6). All cases showed a strong 73-kDa band in both the soluble and insoluble fractions. A strong band at approximately 33 kDa was detected in the sample from the patient diagnosed as having BIBD with chorea, but this band was less prominent in the samples from the other patients.

## Discussion

In the present study, we found that chorea in FTLD patients is related to FUS pathology. Chorea involves continuous movements that are irregular and nonrepetitive, which differ from the repetitive stereotypic behaviors [31] that are present in FTLD patients. These patients lacked muscle tone but show no muscle atrophy. In clinical practice, a combination of various movements is often encountered in a single patient [31, 32]. Determinig the dominant movement type is important in such cases. In our series, chorea was identified as the dominant movement disorder syndrome by each attending doctor during the disease course. Indeed, most HD patients not only exhibit the characteristic chorea, but also display bradykinesia and akinesia [31]. We suggest that clinicians should be aware of the various involuntary movements (including chorea) in treatment of FTLD-FUS patients.

Historically, there has been a general agreement that chorea-like involuntary movements are rare in FTLD [2]. Until recently, few cases of FTLD with chorea have been described, and were considered atypical, as in Pick’s disease [33–36]. One clinical report suggested that chorea is present in FTD patients [37], reporting that two bvFTD cases could be associated with chorea but lack an *HTT* mutation. They further mentioned the potential of a clinical phenotype presenting chorea in FTD, but unfortunately these cases lacked autopsy confirmation of the diagnosis.

To our knowledge, chorea is rarely described in FTLD-tau cases. In patients with TDP-43 mutations, chorea may be present [38, 39]. A patient with a K263E *TARDBP* mutation developed FTD, supranuclear palsy, and chorea, but not ALS, which was associated with TDP-43 accumulation predominantly in subcortical nuclei and the brainstem [40]. More recently, C9ORF72 repeat expansions were reported to be the most common genetic cause of non-HD syndromes [41]. Only two cases of FTLD-FUS with chorea have been previously reported. Lee et al. described one patient with late onset BIBD who was clinically diagnosed with ALS-plus syndrome, and showed diffuse chorea and cognitive dysfunction but no parkinsonism [42]. The second case was described by Yokota et al. [15], and was case 3 in our current study. In our study, we did not detect any FTLD-tau or FTLD-TDP cases with chorea-like involuntary movements.

Chorea is the most common clinical feature in HD, with patients showing severe striatal atrophy. Although the striatum is also severely affected in FTLD-FUS, chorea is considered to be a relatively rare clinical feature in FTLD-FUS, especially compared with HD. In FTLD-FUS, the topographic distribution pattern of the caudate nucleus, nucleus accumbens, and putamen is different from HD. In our series, the head and body of the caudate nucleus is more degenerated than the body and tail, whereas the tail is more degenerated than the body and head in HD [43]. Moreover, the nucleus accumbens is severely degenerated in FTLD-FUS, in contrast to being remarkably preserved in the advanced stage (stage 4) of HD [43, 44]. With the evolution of FTLD-FUS, degeneration in the neostriatum appears to move in a rostro-caudal, ventro-dorsal, and medio-lateral direction.

Chorea is associated with the striatum (caudate nucleus and putamen), globus pallidus, substantia nigra, subthalamic nucleus, and cerebral cortex [43, 45]. Unfortunately, it is difficult to specify the correlation between chorea and our neuropathological findings, since we did not find any significantly different neurodegenerative changes between cases with and without chorea, even in the responsible regions. Differences in the region initially affected or the speed and direction of degeneration may influence the clinical symptoms (including chorea) in FTLD-FUS cases, although more detailed studies are needed to clarify this issue.

As in the pathophysiology of HD, striatal projection neurons of the indirect pathway are vulnerable, while those of the direct pathway are relatively preserved [46]. Severe involvement of striatal projection neurons in both the indirect and direct pathways may explain the rarity of chorea in FTLD-FUS. Alternatively, lesions outside the striatum may cause such a phenotypic difference. The striatum regulates movement through interactions with the cerebral cortex as well as with multiple subcortical nuclei including the globus pallidus, subthalamic nucleus, and some brainstem nuclei. The presence or absence of chorea and related involuntary movements may depend on a delicate functional balance between these structures that form the striatal motor circuits.

Among the FTLD cases in our brain archives, only three patients displayed chorea, and all three patients were diagnosed as having FTLD-FUS with the BIBD subtype. None of the FTLD-tau or FTLD-TDP cases were associated with chorea. Because FTLD-tau and FTLD-TDP comprise the majority of FTLD cases, the paucity of cases with chorea in these groups is remarkable. BIBD is considered to be a generalized variant of Pick’s disease because of its relatively broad distribution of degenerative changes that extend to subcortical structures [47]. The involvement of multiple subcortical nuclei may increase the chances of some BIBD patients developing chorea. It may be noteworthy that BIBD patients without chorea show moderate to severe parkinsonism symptoms in the later stage of disease, whereas those with chorea lack parkinsonism throughout the disease course. Chorea in HD is treated with anti-dopaminergic agents [48]. In BIBD, both the striatum and substantia nigra undergo degenerative changes. Cases with relatively depleted nigral dopaminergic regulation of the striatal motor circuits may be associated with parkinsonism, while those with relatively less severe nigral dysfunction may develop chorea in the absence of parkinsonism. Accordingly, postmortem histopathological analysis of terminal stage lesions may not be sensitive enough to detect such a premortem functional imbalance.

The choreoathetoid movements identified in our series might be influenced by antipsychotic drugs as a risk factor for severe caudate atrophy. Previous studies have stated that chorea in HD is difficult to distinguish from tardive dyskinesia [49]. However, in general, the movements observed in our cases is unlikely to be diagnosed as tardive dyskinesia because of the following points. Tardive dyskinesia is defined in diagnostic criteria as developing due to the use of medications such as antipsychotic drugs (dopamine receptor blocking agents) for more than 3 months, and specifically, dystonia must be present either during ongoing antipsychotic treatment or within 3 months of its discontinuation [50]. In addition, second generation antipsychotics (e.g., olanzapine) rarely cause acute dystonic reactions [51], and tardive dyskinesia might only present when the patients take high-doses [49]. In HD, choreic movements are random, flowing from one part of the body to the other, and frequently superimposed by semi-purposeful movements in an attempt to mask involuntary movements. In contrast, movement in tardive dyskinesia is slow, stereotypic, and repetitive. In cases 1 and 3, we were able to reconfirm such movement features in HD from the clinical records. From our own experience, patients at the onset of tardive dyskinesia predominantly show akathisia and tremor, although choreiform movements may occur. However, this point might reflect a limitation of our study, and further efforts are needed to unveil the association between drug-induced choreoathetoid movements and FTLD-FUS accumulation in diseased conditions.

In our biochemical analyses, a 73-kDa band corresponding to full-length FUS was found at the same intensity in both soluble and insoluble fractions in all cases. This result is inconsistent with a previous report, which showed that the 73-kDa band intensity in the insoluble fraction was stronger in FTLD-FUS cases than normal controls [10]. In the present study, we identified a new FUS fragment of approximately 33 kDa in the insoluble fraction, which was derived from a patient diagnosed as having BIBD with chorea. Because we could not biochemically analyze the BIBD case without chorea, it is unclear whether this fragment is associated with the pathogenic mechanism of BIBD with chorea. However, previous reports show a clear relationship between the band pattern of low molecular weight fragments of insoluble proteins and clinicopathological phenotypes in FTLD-tau [52] and FTLD-TDP [22], suggesting that further biochemical study of insoluble FUS fragments may shed light on FTLD-FUS.

## Conclusions

Our results suggest that choreoathetosis observed in patients with bvFTD could be a clinical marker of the underlying pathology of BIBD. In these cases, severe atrophy of the caudate nucleus and relatively preserved nigral dopaminergic regulation might be associated with chorea in the absence of parkinsonism in BIBD. Further studies are needed to elucidate the exact mechanism by which chorea occurs, which in turn may develop new therapeutic approaches for this incurable condition in FTLD patients.

### Ethics approval and consent to participate

All patients, or in one case in which the patient had died, the next of kin, provided written consent for autopsy and postmortem analyses for research purposes. This study was approved by the ethics committee at the Tokyo Metropolitan Institute of Medical Science, and was performed in accordance with the ethical standards outlined in the 1964 Declaration of Helsinki and its later amendments.

### Consent for publication

Details that might disclose the identity of the participants in this study were omitted.
